# Supplementary material for: Lacticaseibacillus rhamnosus GG in a chewable colonizes the nose and facilitates local immune benefits in allergic rhinoconjunctivitis patients
Source: Microbiol Spectr. 2025 Sep 2;13(10):e00773-25. doi: 10.1128/spectrum.00773-25 (PMC12502598; doi:10.1128/spectrum.00773-25)
Supplement: Supplemental figures, table, and questionnaire — Fig. S1 to S3, Table S1, and questionnaire. [file spectrum.00773-25-s0001.docx]

**Supplementary figures and tables**

***Lacticaseibacillus rhamnosus* GG in a chewable colonizes the nose and facilitates local immune benefits in allergic rhinoconjunctivitis patients.**

Ilke De Boeck^1*^, Irina Spacova^1*^, Eline Cauwenberghs^1^, Tom Eilers^1^, Thies Gehrmann^1^, Karlien Van den Bossche^2,3^, Katleen Martens^4^, Sandra Condori-Catachura^1^, Kato Michiels^1,5^, Fien De Winter^6^, Samir Kumar-Singh^6^, Nicolas Bruffaerts^7^, Ann Packeu^7^, Peter W. Hellings^4,8^, Anneclaire Vroegop^2,3^, Klara Van Gool^9^, Olivier M. Vanderveken^2,3^, Sarah Lebeer^1$^


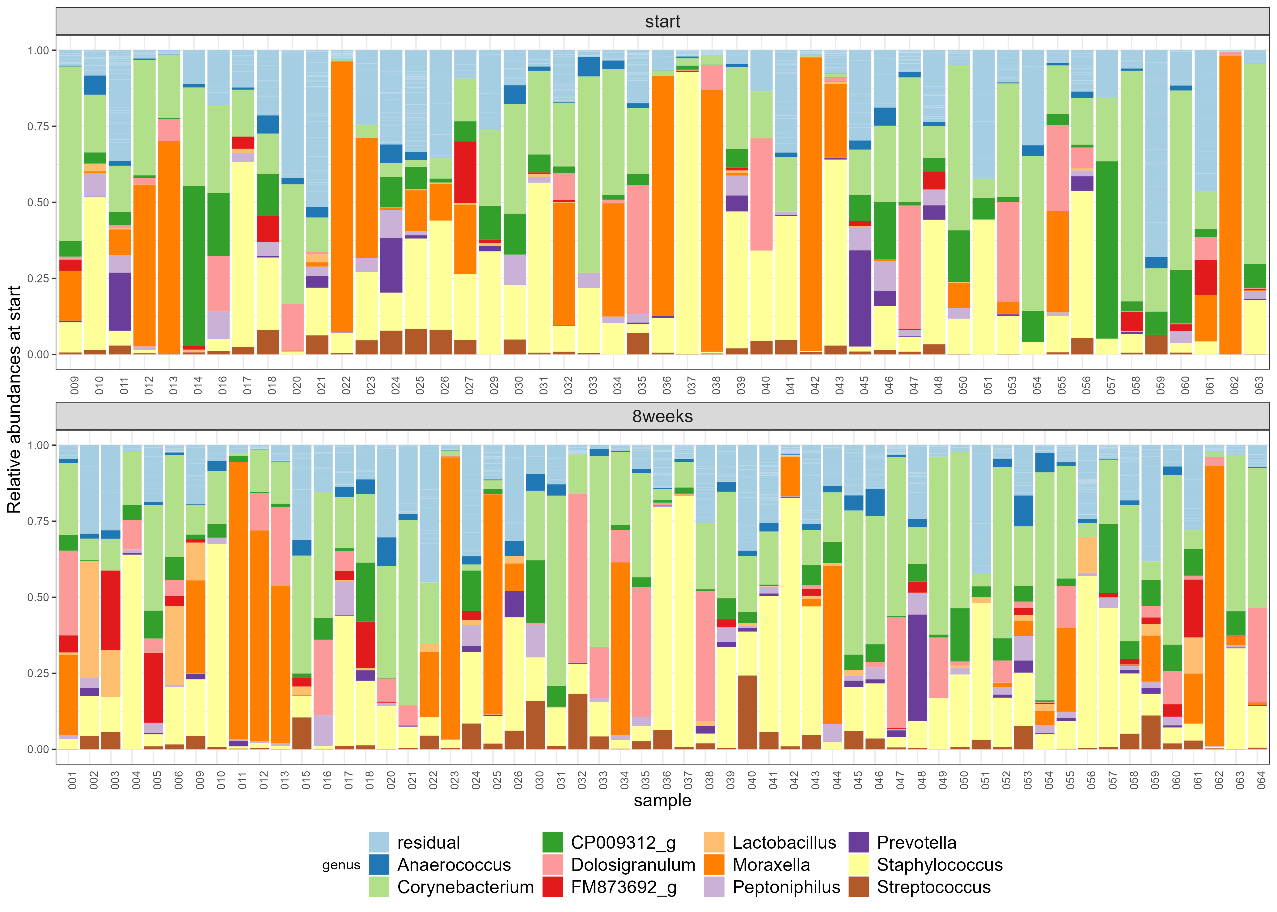


Figure S1: Microbiome profiles in the nasopharynx at start and end of the study period. The eleven most abundant genera are shown.


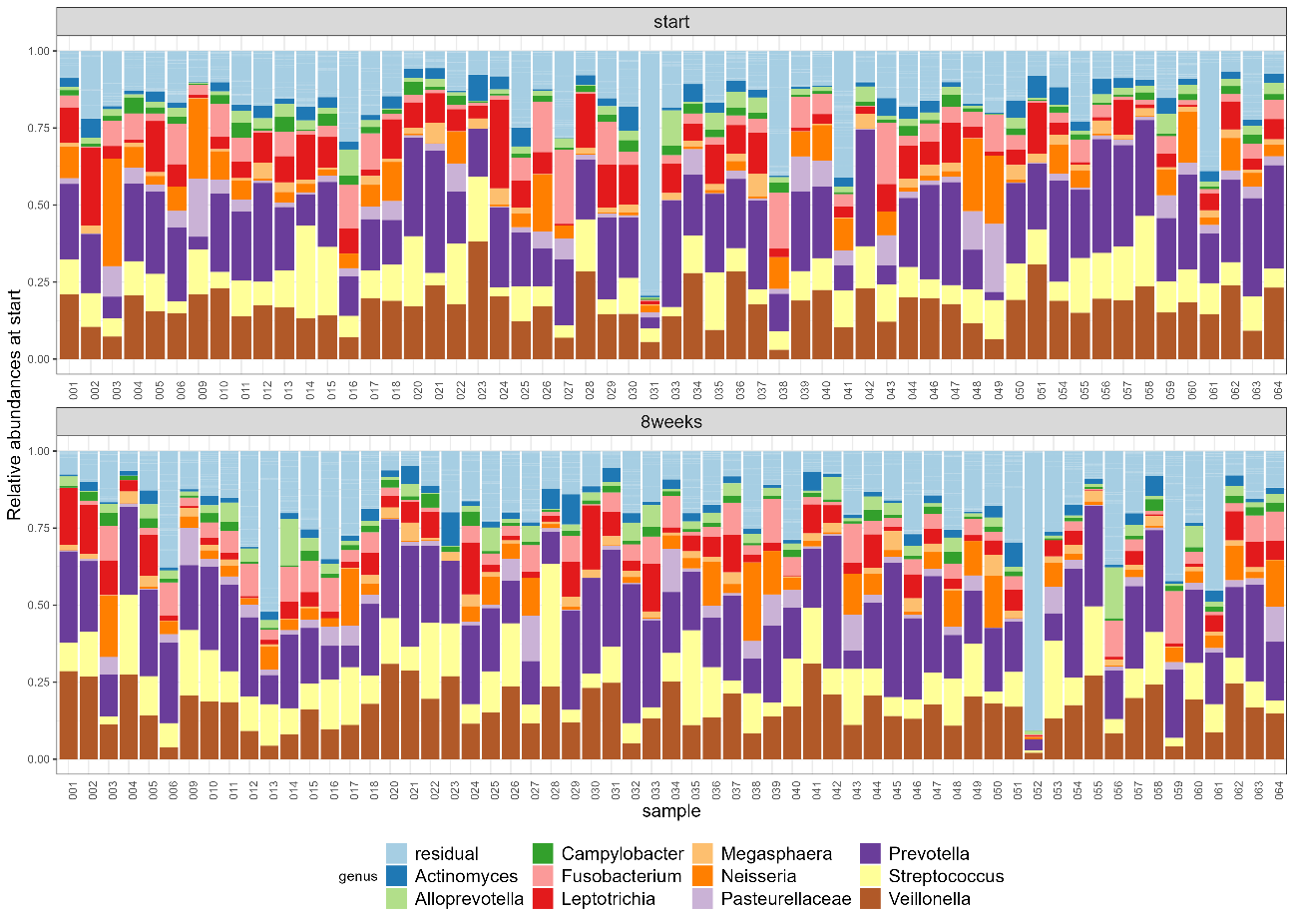


Figure S2: Microbiome profiles in the oropharynx at start and end of the study period. The eleven most abundant genera are shown.


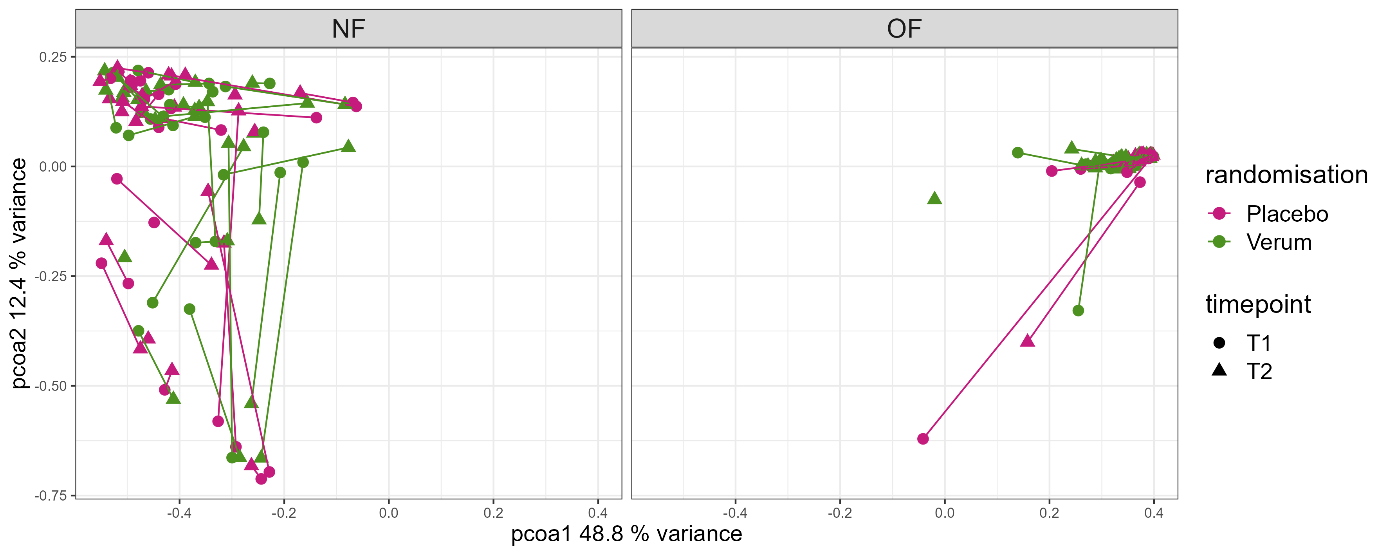


Figure S3: Principal Coordinate Analysis (PCoA) to visualize the diversity of taxa before and after the intervention in the nasopharynx (NF) and oropharynx (OF).

Table S1: Medication use in the participants at start (one timepoint), and during the intervention period (daily monitored throughout the study). The use of anti-histamine medication and/or corticoid sprays is shown shown as a percentage over the study.

| Participant | Treatment | Timepoint | Frequency_anti-histamine | Frequency_spray |
| --- | --- | --- | --- | --- |
| DSM001 | B | start | Only when complaints | NA |
| DSM001 | B | during study | 18% | 0% |
| DSM002 | B | start | Daily use | Daily use |
| DSM002 | B | during study | 45% | 36% |
| DSM003 | A | start | NA | NA |
| DSM003 | A | during study | 0% | 0% |
| DSM004 | B | start | Only when complaints | Only when complaints |
| DSM004 | B | during study | 30% | 0% |
| DSM005 | A | start | Daily use | NA |
| DSM005 | A | during study | 0% | 0% |
| DSM006 | B | start | Daily use | NA |
| DSM006 | B | during study | 5% | 0 |
| DSM009 | A | start | Only when complaints | NA |
| DSM009 | A | during study | 29% | 0 |
| DSM010 | A | start | Only when complaints | NA |
| DSM010 | A | during study | 59% | 0 |
| DSM011 | B | start | NA | NA |
| DSM011 | B | during study | 0 | 0 |
| DSM012 | B | start | Only when complaints | NA |
| DSM012 | B | during study | 29% | 0 |
| DSM013 | A | start | Daily use | Daily use |
| DSM013 | A | during study | 60% | 57% |
| DSM014 | A | start | Only when complaints | Only when complaints |
| DSM014 | A | during study | 46% | 28% |
| DSM015 | A | start | Daily use | NA |
| DSM015 | A | during study | 2% | 0 |
| DSM016 | B | start | Only when complaints | NA |
| DSM016 | B | during study | 5% | 0 |
| DSM017 | B | start | Only when complaints | Only when complaints |
| DSM017 | B | during study | 4% | 0 |
| DSM018 | B | start | Daily use | Only when complaints |
| DSM018 | B | during study | 58% | 0 |
| DSM020 | A | start | Only when complaints | NA |
| DSM020 | A | during study | 33% | 33% |
| DSM021 | A | start | Daily use | Only when complaints |
| DSM021 | A | during study | 33% | 18% |
| DSM022 | A | start | Only when complaints | NA |
| DSM022 | A | during study | 7% | 0 |
| DSM023 | B | start | NA | NA |
| DSM023 | B | during study | 0 | 0 |
| DSM024 | A | start | Only when complaints | NA |
| DSM024 | A | during study | 96% | 0 |
| DSM025 | B | start | Daily use | NA |
| DSM025 | B | during study | 0 | 0 |
| DSM026 | B | start | Only when complaints | Only when complaints |
| DSM026 | B | during study | 0 | 16% |
| DSM027 | A | start | Only when complaints | NA |
| DSM027 | A | during study | 16% | 0 |
| DSM028 | B | start | Daily use | NA |
| DSM028 | B | during study | 0 | 0 |
| DSM029 | A | start | NA | NA |
| DSM029 | A | during study | 0 | 0 |
| DSM030 | B | start | Only when complaints | Only when complaints |
| DSM030 | B | during study | 0 | 0 |
| DSM031 | A | start | Only when complaints | NA |
| DSM031 | A | during study | 0 | 0 |
| DSM032 | B | start | Only when complaints | Only when complaints |
| DSM032 | B | during study | 18% | 0 |
| DSM033 | B | start | Only when complaints | NA |
| DSM033 | B | during study | 0 | 0 |
| DSM034 | A | start | NA | NA |
| DSM034 | A | during study | 0 | 0 |
| DSM035 | A | start | Only when complaints | NA |
| DSM035 | A | during study | 20% | 0 |
| DSM036 | A | start | Only when complaints | Only when complaints |
| DSM036 | A | during study | 44% | 0 |
| DSM037 | B | start | Only when complaints | Only when complaints |
| DSM037 | B | during study | 43% | 0 |
| DSM038 | B | start | Only when complaints | NA |
| DSM038 | B | during study | 79% | 0 |
| DSM039 | A | start | Daily use | NA |
| DSM039 | A | during study | 2% | 0 |
| DSM040 | B | start | Daily use | NA |
| DSM040 | B | during study | 2% | 0 |
| DSM041 | B | start | Only when complaints | NA |
| DSM041 | B | during study | 9% | 2% |
| DSM042 | A | start | Daily use | NA |
| DSM042 | A | during study | 0 | 0 |
| DSM043 | B | start | Only when complaints | NA |
| DSM043 | B | during study | 32% | 0 |
| DSM044 | A | start | Only when complaints | NA |
| DSM044 | A | during study | 18% | 0 |
| DSM045 | A | start | Daily use | Daily use |
| DSM045 | A | during study | 0 | 0 |
| DSM046 | A | start | Daily use | Only when complaints |
| DSM046 | A | during study | 21% | 7% |
| DSM047 | B | start | NA | Only when complaints |
| DSM047 | B | during study | 0 | 0 |
| DSM048 | B | start | NA | Only when complaints |
| DSM048 | B | during study | 0 | 67% |
| DSM049 | A | start | NA | NA |
| DSM049 | A | during study | 38% | 2% |
| DSM050 | B | start | Daily use | Only when complaints |
| DSM050 | B | during study | 25% | 25% |
| DSM051 | B | start | Daily use | Daily use |
| DSM051 | B | during study | 34% | 0 |
| DSM052 | B | start | NA | Daily use |
| DSM052 | B | during study | 0 | 36% |
| DSM053 | B | start | Daily use | NA |
| DSM053 | B | during study | 2% | 0 |
| DSM054 | A | start | Only when complaints | NA |
| DSM054 | A | during study | 4% | 0 |
| DSM055 | A | start | Only when complaints | NA |
| DSM055 | A | during study | 0 | 0 |
| DSM056 | B | start | Only when complaints | NA |
| DSM056 | B | during study | 33% | 0 |
| DSM057 | B | start | Only when complaints | NA |
| DSM057 | B | during study | 0 | 0 |
| DSM058 | A | start | NA | NA |
| DSM058 | A | during study | 0 | 0 |
| DSM059 | B | start | Daily use | Daily use |
| DSM059 | B | during study | 71% | 22% |
| DSM060 | A | start | NA | NA |
| DSM060 | A | during study | 0 | 0 |
| DSM061 | B | start | NA | NA |
| DSM061 | B | during study | 3% | 0 |
| DSM062 | A | start | Only when complaints | Only when complaints |
| DSM062 | A | during study | 5% | 0 |
| DSM063 | A | start | Only when complaints | NA |
| DSM063 | A | during study | 25% | 0 |
| DSM064 | A | start | Only when complaints | NA |
| DSM064 | A | during study | 29% | 0 |

**General Questionnaire**

*This questionnaire Nods to be filled in at the start of the study*

Age? ………… year

Gender:

- Woman
- Man
- Transman
- Transwoman
- Other

When did your nasal complaints first started?

………………………………………………………………………………………………………………………………………………………………………………………………………………………………………………………………………………………………………………………………………………………………………………………………………………

Describe your profession

………………………………………………………………………………………………………………………………………………………………………………………………………………………………………………………………………………………………………………………………

Do your symptoms increase during or after performing your job?

- No
- Yes

Do you smoke?

- No
- Yes, how many? …………………………………………………………………………………………
- Ex-smoker since ……………………………………………………………………………………

Have you ever received any of the following diagnoses related to allergy? Multiple answers are possible.

- - Allergic asthma
  - Hay fever/inhalation allergy
    - Tree pollen allergy
    - Grass pollen allergy
    - Housedust mite allergy
    - Allergy to animal(s) + which one (s)? ............................
    - Other inhalation allergy:.........................
  - Eczema
  - Food allergy to …………………..........................
  - Other (medication, insects, contact allergy): ………………….

Do you take any medication against your allergy?

- No
- Yes

Which? ………………………………………………………………………………………

Frequency? ………………………………………………………………………………

How? □ Daily

□ Only when experiencing complaints

Did you have any antibiotic treatment in the past 5 years? …………………………………………………

When was your last antibiotic treatment?

…………………………………………………

Do you use probiotics or good bacteria?:

- In dairy products such as Yakult, Actimel?

□ No

□ Yes

How often ………………………

Which products ……………………….....................................................

Did you consume these products the past week? ………………………

- In capsules such as Enterol, Probactiol or other supplements available at the pharmacy?

□ No

□ Yes

How often ………………………

Which products ……………………….....................................................

Did you consume these products the past week? ………………………

How would you describe the area where you grew up? This question refers to the period of your childhood during which you stayed the longest, in order to assess possible influences of urbanization.

- City center
- Village center
- Residential area
- Busy road
- Rural area
- Green zone/recreational area/...
- Industrial zone

How would you describe the area where you currently live?

- City center
- Village center
- Residential area
- Busy road
- Rural area
- Green zone/recreational area/...
- Industrial zone

How many hours per day do you spend outside? …………………..

Do you come into contact with... on a daily basis?

- A great deal of greenery
- A lot of greenery
- A fair amount of greenery
- Little greenery
- Very little greenery
- No greenery

What is your main mode of transportation?

- Walking
- By bike
- Motor / scooter
- Car
- Public transport
- Other: …………………….

How often do you have a cold or flu-like symptoms?

- Never
- 1-2x a year
- 3-5x a year
- 6-10x a year
- >10x a year
- I don’t know

Do you have contact with animals at home or at work?

- Yes,
- At work (how many animals + which animals)?..................................................
- I have pets,
- Inside, how many and which animals?............................................
- Outside, how many and which animals?............................................
- No

How often do you eat or drink the following products?

|  | **Multiple times per day** | **Daily** | **>3x/week** | **Weekly** | **Monthly** | **Seldom** | **Never** |
| --- | --- | --- | --- | --- | --- | --- | --- |
| Dairy products (cheese, milk, yoghurt, …) |  |  |  |  |  |  |  |
| Fermented foods (olives, salami, sauerkraut, sourdough bread, ... ) |  |  |  |  |  |  |  |
| Alcohol |  |  |  |  |  |  |  |
| Meat |  |  |  |  |  |  |  |
| Animal products (eggs, cheese, gelatin, etc.) |  |  |  |  |  |  |  |
| Fish |  |  |  |  |  |  |  |
| Sweet drinks with sugar (e.g. soft drinks, fruit juice) |  |  |  |  |  |  |  |
| Sweet drinks without sugar (e.g. diet soda) |  |  |  |  |  |  |  |
| Fruit |  |  |  |  |  |  |  |
| Vegetables |  |  |  |  |  |  |  |

Are you following a special diet? (Select all that apply)

- No
- Vegetarian
- Vegan
- Low carbs
- Other: …………………………………………..

Do you have one of the following conditions? (Please state the condition)

| Systemic  Eg. rheumatism, artritis, MS, … |  |
| --- | --- |
| Skin  Eg. Psoriasis, acne, … |  |
| Heart/blood vessels  Eg. endocarditis, aneurysma, … |  |
| Gatsrointestinal  Eg. Crohn’s disease, colitis, … |  |
| Ear-Nose-Throat  Eg. chronic rhinosinusitis |  |
| Airways  Eg. asthma, COPD, … |  |
| Eyes  Eg. Glaucoma, cataract,... |  |
| Hormonal  Eg. Thyroid or diabetes (type 1/type 2) |  |
| Reproductive system  Eg. Endometriose |  |
| Blood  Eg. Anemia, leukemia |  |
| Other |  |

If you had to take medication in the past three months, please complete this overview

| **Name** | **Dose** | **Start date** | **Stop date** | **Reason** |
| --- | --- | --- | --- | --- |
|  |  |  |  |  |
|  |  |  |  |  |
|  |  |  |  |  |
|  |  |  |  |  |
|  |  |  |  |  |
|  |  |  |  |  |
|  |  |  |  |  |
|  |  |  |  |  |
|  |  |  |  |  |
|  |  |  |  |  |
|  |  |  |  |  |
|  |  |  |  |  |
|  |  |  |  |  |
|  |  |  |  |  |

Are your nasal complaints (put a cross next to the correct answer)

- Always present
- Provoked by (select all that apply)

□  Temperature changes

□  Certain smells (parfum, smoke)

□  Physical activities

□  Emotional stress

□  Changes in humidity

□ Allergens. If yes, which ones? ………………………………………………………………………

□ Other factors? ………………………………………………………………………

- I don’t know?
